# Advancements in the Management of Pediatric and Adult Inflammatory Bowel Disease: A Systematic Review of Treatment Strategies and Long-Term Outcomes

**DOI:** 10.7759/cureus.72324

**Published:** 2024-10-24

**Authors:** Victor O Adedara, Charles A Adedara, Nicola D Ruth, Grant U Alozie, Nate Nettagul

**Affiliations:** 1 Medicine, St. George's University School of Medicine, St. George, GRD; 2 Family Medicine, University of Mississippi Medical Center, Mississippi, USA; 3 Neonatology, Dudley Group NHS Foundation Trust, Dudley, GBR; 4 Pediatric Gastroenterology, Dudley Group NHS Foundation Trust, Dudley, GBR; 5 Cardiothoracic Surgery, St. George's University School of Medicine, St. George, GRD; 6 Otolaryngology, St. George's University School of Medicine, St. George, GRD

**Keywords:** adult ibd, biologic therapies, crohn’s disease, inflammatory bowel disease, long-term outcomes, pediatric ibd, personalised medicine, small molecules, treatment outcomes, ulcerative colitis

## Abstract

Inflammatory bowel disease (IBD), including Crohn's disease and ulcerative colitis (UC), remains a clinically complex condition in children and adults. This study is a systematic analysis of key developments in the treatment of inflammatory bowel diseases, as well as their efficacy and safety over time. Early diagnosis of pediatric IBD is very important since it affects growth and development in children. New therapeutic approaches like biological agents, small molecules, and gene or targeted drugs have given the medical fraternity new treatment protocols. There is a trend towards more selective therapies for adult IBD, especially for anti-tumor necrosis factor (anti-TNF) biologics, integrin antagonists, and interleukin-12/23 (IL-12/23) inhibitors. This review emphasizes the need for patient management where early intervention leading to mucosal healing has been identified to predict durable outcomes. Systematic analysis of existing literature comparing childhood and adult populations shows that morbidity, pathophysiology, therapeutic outcome, as well as the potential for adverse outcomes are dissimilar, which supports the need for differentiated therapy. This work also looks at long-term consequences of the intervention course, the avoidance of surgery, and an improvement in the quality and stability of life as well as reduction in further development of malignant transformation. The new developing strategy of gut microbiome modification and nutrition support for maintaining remission is also argued. Despite these progresses, issues still persist concerning the effectiveness of treatments, side effects, and patients’ compliance. These recommendations give this review a prospective outlook of treatment regimens likely to define the future of IBD management for all age groups.

## Introduction and background

Background and context

Inflammatory bowel disease (IBD) is defined as a chronic autoimmune condition that is characterized by the inflammation of the bowl with symptoms including severe belly pain and chronic diarrhea. Crohn’s disease (CD) and ulcerative colitis (UC) are two distinct types of inflammatory bowel diseases. CD can affect any part of the gastrointestinal tract, often involving deeper layers of the bowel wall, while UC is limited to the colon and typically affects only the innermost lining of the colon [1]. Both conditions are defined as inflammation of the gastrointestinal (GI) tract and have several debilitating severe manifestations, such as abdominal pain, diarrhea, rectal bleeding, weight loss, and fatigue [2]. The increasing rates of IBD in various populations worldwide have stimulated studies that aim to identify the mechanisms by which the diseases occur and ways to manage the diseases [3].

In the past few decades, there has been a shift in the IBD therapeutic services offered. Conventional therapies like corticosteroids and immune-modulators are increasingly being or have been supplemented by the modern targeted therapeutic agents [4,5,6]. Anti-TNF biologics have emerged as the primary treatment options since they provide a better possibility of inflammation management and improve patient progress. Moreover, new medications, including integrin inhibitors, Janus kinase inhibitors, and small molecules, have been added to the available therapies, predominantly for patients who do not benefit from conventional treatments [7,8].

In pediatric and adult patient follow-up, dose density is often a marker of therapeutic efficacy, as targeted treatment is administered to minimize treatment-related toxicity such as infection, malignancy, or drug toxicity [9,10]. The purpose of the present systematic review is to synthesize the most recent literature on the topic, comparing novel therapeutic modalities in both children and adults, illustrating their effectiveness, side effects, and durability, and evaluating novel trends of IBD treatment, including but not limited to individualized medicine and microbiome-targeted interventions.

Research Question

The research question is to find out the different types of treatment available for children and adults with IBD and their impact on the general prognosis of the disease, including the rates of relapse, the quality of life, and complications.

Research Aim

This research aims to comprehensively review the published literature on managing pediatric and adult IBD to determine optimal therapeutic approaches and explore their effectiveness in achieving sustained disease control, improving the overall quality of life, and preventing adverse effects.

Research Objectives

The following objectives guide the achievement of effective results. The study aims to assess the effectiveness of treatments currently used for IBD, including biologics, small molecules, and conventional medications in pediatric and adult patients. Additionally, it seeks to evaluate the efficacy, safety, and necessity of these treatments regarding the remission of the disease, the prevention of surgery, and malignancy, which would be related to the outcome. The study also explores the potential impact of innovative therapeutic strategies like personalized medicine and microbiome-based therapy in IBD patients. It also seeks to determine the distinct patterns of disease manifestation, course, and prognosis between children and adult patients and the rationale for distinct therapeutic approaches.

## Review

Methods 

In this study, a systematic review was employed to assess various novel approaches to the treatment of IBD in both children and adults. The study followed the PRISMA checklist for conducting the systematic review. A thorough search plan was utilized to find relevant studies published in peer-reviewed journals. The process involved defining the research question, selecting databases to search for articles, establishing inclusion and exclusion criteria, screening the articles, and extracting data for synthesis and analysis.

The inclusion and exclusion criteria were refined to ensure a concise data set. A list of keywords was generated and used to retrieve the most relevant research. These keywords included inflammatory bowel disease, Crohn’s disease, ulcerative colitis, pediatric IBD, adult IBD, biological therapies, small molecules, treatment outcomes, long-term outcomes, personalized medicine, and microbiome therapy. Boolean operators were used to narrow the search further. The literature search focused on articles published between January 2014 and September 2024 to capture recent advancements in biologics, small molecules, target therapies, and precision medicine.

Boolean search terms were utilized to improve the specificity and effectiveness of the search results. Using operators like AND and OR, the search could be generalized or narrowed down to address specific research questions. The databases chosen for the literature search included Google Scholar, PubMed, Medline, Cochrane Library, Embase, and Scopus. These databases were selected for their high impact on gastroenterology and IBD research. Only studies published in English were included due to the researchers' limitations in understanding other languages, which could impact the quality of the results.

**Figure 1:**
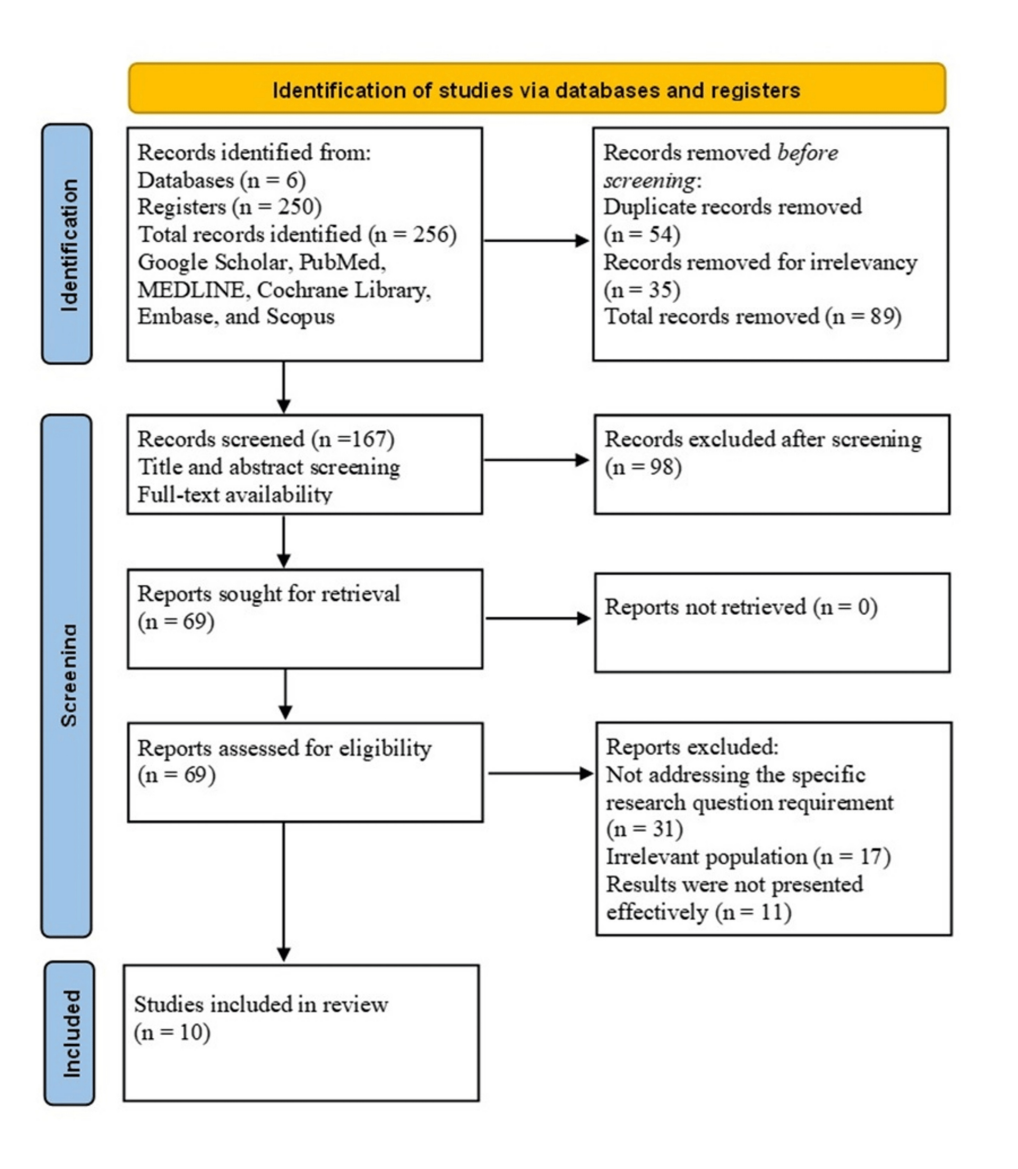
Study selection process for systematic review using PRISMA guidelines The figure was created by the authors

The exclusion criteria eliminated animal studies, case reports, studies not specific to IBD or not addressing treatment outcomes, and studies with improper research methodology. Reviews and editorials were also excluded. A systematic approach was adopted for data extraction and quality assessment, guided by the PRISMA flow diagram to extract meaningful data for the research in Figure 1. 

The findings of the included studies were synthesized using qualitative data synthesis (QDS). Data extraction was performed on a pre-designed form, collecting characteristics such as participants, interventions, comparisons, outcomes, and other qualitative data. The synthesis aimed to extract common ideas and trends regarding the effectiveness, safety, and further evolution of existing and novel treatment approaches, biologics, small molecules, and other drug classes based on clinical data. Qualitative synthesis was beneficial in comparing treatment approaches between children and adults and identifying research areas requiring further information.

The data collected from the selected articles were analyzed qualitatively using thematic analysis, which seeks to find recurring patterns within the data. The thematic analysis allows data to be sorted and standardized, making analyzing information based on thematic distributions easier. The major themes identified included the perceived effectiveness of treatments, sustained management of conditions, improvements in quality of life, and side effects. Secondary themes discussed include early intervention, individualized recommendations, and the importance of the gut microbiome. These themes were then used to draw relevant conclusions regarding the treatment progress for IBD and how these relate to differences between pediatric and adult patients.

Results 

This section explores the findings from the research articles used to evaluate the research question. The summary of each research paper’s findings is presented in Table 1. 

**Table 1:** Summary of recent research on treatments and management strategies for inflammatory bowel disease. The table was created by the authors. IBD: inflammatory bowel disease, UC: ulcerative colitis, JAK: janus kinase, S1P: sphingosine 1-phosphate

Author(s)	Year	Title	Methodology	Summary of Findings
Green et al., [11]	2023	Recent developments in the assessment and management of inflammatory bowel disease in childhood.	Review of pediatric IBD management strategies.	The article demonstrates the advances in diagnostics, such as endoscopy and genetic testing of pediatric IBD. The guide stresses early intervention with biological agents and nutrition therapy in a child’s treatment process to enhance outcomes in disease remission as well as growth dimensions among the children. There are indications of the requirement for treating patients with individualized care plans to cater to the disease’s prognosis and stages.
Ungaro et al., [12]	2019	A treat-to-target update in ulcerative colitis: A systematic review	A systematic review of treat-to-target strategies in ulcerative colitis.	This research shows that treat-to-target approaches, where patients are targeted for clinical and endoscopic remission, enhance the disease’s long-term management in UC. These approaches also incorporate biomarkers and constant serial monitoring to enhance treatment outcomes and avert issues, including surgery.
Foppa et al., [13]	2023	Microbiota and IBD: current knowledge and future perspectives.	Literature review on microbiota’s role in IBD and future research perspectives.	This paper explores the details of the processes that make up the gut microbiota in IBD and presents microbial-based treatment methods such as FMT and probiotics. Novel interventions addressing the microbiome appear to have potential. However, there is a need for increased trials to help determine their effectiveness in the long term.
Oliveira et al., [14]	2017	Diagnosis and management of inflammatory bowel disease in children	The review article focused on pediatric IBD diagnosis and management practices.	In this article, several points are made concerning the role of early diagnosis and a multimodal approach to the treatment of children with IBD. In treatment, the document emphasizes corticosteroids, immunomodulators, and biological agents but underlines the importance of vigilance for growth, nutrition, and psychosocial aspects for an optimum result.
Xu et al., [15]	2022	Current status of novel biologics and small molecule drugs in the individualized treatment of IBD	Literature review of novel biologics and small molecule treatments in IBD.	The study explores emerging biologics, such as IL-12/23 inhibitors and JAK inhibitors, as effective treatment options in IBD patients who have poor prognosis during conventional management of IBD. The evidence indicates that these therapies decrease the prescription rate of steroids and surgeries as well as delay treatment relapse, especially in treatment-refractory patients.
Park et al., [8]	2022	Personalized medicine in inflammatory bowel disease: Perspectives on Asia	Review on personalized medicine in IBD treatment, focusing on Asia.	This study focuses on the role of genetics and environment in the Asian context when managing and treating IBD. Targeted therapy, particularly pharmacogenomics, is evolving in various IBD subgroups to improve both the efficacy of therapy and the reduction of undesired side effects.
Zenlea et al., [16]	2014	Immunosuppressive therapies for inflammatory bowel disease	Review of immunosuppressive treatments for IBD.	The article discusses the application of immunosuppressive drugs such as thiopurines and methotrexate in sustaining remission of IBD patients. Despite its value for controlling diseases, the long-term outcomes have some concerns and risks, such as infection and malignancy.
Kurowski et al., [17]	2020	Differences in biologic utilization and surgery rates in pediatric and adult Crohn’s disease	Cohort study analyzing biologic usage and surgery rates in pediatric vs. adult Crohn’s disease.	This study shows that pediatric Crohn’s disease patients are more likely to receive biological treatment and less likely to undergo surgery than adult patients. Health workers recommend that parents start their children on biologics as early as possible, as this helps prevent the illness from worsening, and there will be little need for surgery among young patients.
Buono et al., [18]	2022	Sphingosine 1-phosphate modulation in inflammatory bowel diseases	Literature review on sphingosine 1-phosphate (S1P) receptor modulators in IBD treatment.	This paper explores the role of S1P receptor modulators, including ozanimod, in treating IBD, where it acts in the exclusion of lymphocytes in the intestines. Such modulators offer relief from inflammation, have the potential to prevent relapses, and might be used as prospective alternatives to biologics for patients with UC and CD.
Gubatan et al., [19]	2021	Anti-Integrins for the treatment of inflammatory bowel disease: current evidence and perspectives	Review of anti-integrin therapies for IBD.	The article explains anti-integrin agents, including vedolizumab, applied to treat moderate to severe IBD. Anti-integrins influence leukocyte trafficking, thus providing selective inflammation regulation. These therapies help prevent relapse, and there have been relatively fewer side effects when compared to conventional immunosuppressive drugs.

Discussion

This systematic review highlights critical advancements in the care of both pediatric and adult IBD, with a particular focus on recent management strategies and their outcomes [20]. A key finding is the growing emphasis on early detection and intervention, especially in children. Emerging diagnostic tools, such as genetic testing and endoscopic evaluations, play a pivotal role in improving the treatment of pediatric IBD [11,14]. In particular, the timely and intense use of biological medications during the early stage of pediatric IBD is crucial in determining disease remission and growth [21]. Biologic drugs have become core treatment strategies, shifting from conventional small-molecule medicine to novel, early-intervention treatments for pediatric patients.

When it comes to adult IBD, one of the current approaches proposed is the treat-to-target (T2T) design. The study shows that focusing on clinical and endoscopic remission is superior and enhances long-term disease-specific outcomes. It is the best approach to handling diseases like ulcerative colitis (UC) [12]. Controlling biomarkers and adjusting them with specific parameters, in particular cases, helps tailor the therapy to the patient and increases the accuracy of medication consumption [22,23].

Other significant areas of innovation are emerging microbiome-based treatments. Microbiota modulations analyzed in the context of microbiota-targeting interventions include fecal microbiota transplantation and probiotics. The microbiome is critical in pathogenesis and inflammatory bowel disease progression. Dysbiosis in the gut microbial community has been linked to increased inflammation and disease activity in IBD patients [13]. While these therapies are still in their experimental stage and undergoing test trials, they can potentially change the nature of IBD treatments as they target the fundamental dysbiosis in the gut microbiota.

New classes of biologics and small molecules, such as Janus kinase (JAK) inhibitors and IL-23 inhibitors, hold promise for moderate to severe IBD patients, especially those who are primary non-responders [24]. The study elaborates that these therapies enhance remission and ameliorate inflammation in treatment-refractory conditions. Another remarkable advancement is the employment of anti-integrin treatments, which act principally on the site of the inflamed gut wall and exhibit fewer systemic side effects than immunosuppressive drugs [19].

Moreover, the issue of the long-term safety of immunosuppressive treatments and the future application of such therapies is still raised. It stresses the necessity of surveillance throughout the treatment course due to higher infection and cancer rates related to agents such as thiopurines and methotrexate [16]. This stresses optimizing efficiency and minimizing detrimental effects for IBD patients [25].

Lastly, progress in IBD management has changed the treatment approach significantly more in the present era because of the introduction of biomarkers, personalized medicine, and associated targeted biologics that enable better disease modulation and ultimate patient outcomes. The diagnostics and status of inflammatory and serum biomarkers, such as C-reactive protein (CRP) and fecal calprotectin, are essential in monitoring disease activity and guiding treatment decisions in IBD. These biomarkers provide valuable insights into the severity of inflammation, helping clinicians assess treatment efficacy and adjust therapies as needed. Moreover, regularly evaluating these biomarkers can predict disease flares and complications, ultimately improving patient management and outcomes [26,27]. Presumably, the future interest will again be aimed at further refining and enhancing these approaches to achieve the best outcomes and eliminate potential adverse effects.

## Conclusions

The systematic review of the current literature reveals significant advancements in the diagnosis and management of pediatric and adult IBD over the last decade. Biologics, particularly in children and young patients, have been pivotal in achieving disease remission, emphasizing the importance of early and intensive management. These developments underscore the need for early detection and the implementation of treatment plans that improve prognosis. Introducing T2T approaches and biologics has significantly enhanced patients' quality of life and disease remission rates. Furthermore, new therapeutic classes, including microbiota-targeted therapies, JAK inhibitors, and IL-23 inhibitors, align with the shift toward precision medicine, offering promising alternatives for IBD treatment.

While these approaches hold substantial promise, additional long-term studies are necessary to evaluate the effectiveness and safety of these treatments over extended periods. Moreover, further research into the underlying causes of IBD will provide a deeper understanding of the disease and enable more precise interventions. Overall, this review highlights the ongoing need for innovation and research to improve both short and long-term outcomes for IBD patients, with an increasing focus on patient-centered care beyond competent management.
